# Department of Practice of Medicine

**Published:** 1894-04

**Authors:** 

**Affiliations:** Professor of Pathology and Lecturer on Mental and Nervous Diseases in the Medical Department of the University of Texas, Galveston


### DEPARTMENT OF PRACTICE OF MEDICINE.

EDITED BY PROF. ALLEN J. SMITH, M. D., GALVESTON,
Professor of Pathology and Lecturer on Mental and Nervous Diseases in the Medical Department of the University of Texas, Galveston.

THE TREATMENT OF DIPHTHERIA.—Dr. Arthur Weber, of New Orleans (*N. O. Med. and Surg. Journ.*, February, 1894), has recently reported a series of eighteen cases of diphtheria, treated by the following routine method, with recovery in all but one case. The throat is sponged every twenty minutes during the day, and every hour during the night, with pure peroxide of hydrogen (Marchand's) until the membrane has disappeared. Thereafter a spray is continued every two or three hours for several days. When peroxide of hydrogen is too irritating to the throat, it may be somewhat diluted with water and slightly alkalinized. At each sitting it is necessary that the parts involved be well attended to; never introduce the nozzle only two or three times, but continue the application for a minute or two, permitting the child to breathe after each application. At the commencement of each case, one-twentieth grain of calomel with one grain of bicarbonate of sodium should be given until there is an increase in the salivary secretion and production of four or five characteristic calomel stools, usually within 24 or 48 hours. The following prescription, given in fluid drachm doses every two or three hours, is to be administered after the action of the calomel:

℞ Tincture of chloride of iron...........fl ʒiss.

Glycerine..............................flʒ iv.

Water...............................fl ℥ ii.

For any indication of heart failure, small doses of strychnine and dignitalis are given. As food, brandy, milk-toddies, egg-nog, white of egg beaten up in water, the juice of meat broiled very rare, and cocoa are ordered at regular intervals.

---

RECOVERY FROM DIABETIC COMA BY ADMINISTRATION OF OXYGEN GAS.—Dr. L. W. Bickle (*Am. Med. Surg. Bull.*, December, 1893), in a critical and clinical article upon diabetes records the following interesting case: A lady of 67 years of age, suddenly lost her memory for words one day (July 17, '92) after dinner. Her mind wandered, and she presently became unconscious. Five hours later the unconsciousness was complete, temperature subnormal, pulse irregular, intermittent and feeble, breathing somewhat stertorous, pupils equal and no evident paralysis, uneasy movements from side to back and to the side. She could be aroused with some difficulty, but did not then recognize her relatives, and muttered incoherently. Examination of her urine showed the presence of large proportions of sugar. A dose of ten grains (.6 gram.) of calomel was placed upon her tongue, and a mixture of morphine, ammonia and ether was administered, both without effect in two hours; then she was made to inhale oxygen gas. In five minutes there were signs of returning consciousness, and in five more she opened her eyes, recognized her relatives and spoke rationally. She was then allowed to sleep, and in the morning was quite conscious again. The oxygen was made in a retort by the bedside, and after passing through a wash-bottle of water, was administered through a long rubber tube passed into the mouth. Some months later, at the date of the writing, she was doing well on a partially restricted diet of milk, meat, green vegetables, with half her usual amount of bread, and saccharine instead of sugar.

---

THE TREATMENT OF SPASMODIC TORTICOLLIS BY CONIUM. —Dr. Wharton Sinkler (*Med. and Surg. Reporter*, January 13, 1894), records two cases of spasmodic wry-neck entirely relieved by the administration of conium. He recommends the fluid ex tract of the seeds, and usually begins with fifteen or twenty drops. Besides having seen beneficial results in these cases from such large doses of the remedy, the writer states that in several instances of painless muscular spasm in other positions, he has met with similar beneficial results from conium.

---

INFANTILE SCORBUTUS.—Dr. H. L. Taylor (*Am. Med. Surg. Bull.*, February 1, 1894), writes of a case of scurvy appearing in a female infant of eleven and one-half months of age. The child has been fed almost, if not quite from birth, on a particular brand of condensed milk, which had appeared to agree with her, although for a short time the passages had been rather stringy in character. The child had been nursing less and less, the jaws becoming sore, and the breath heavy. She was restless and sleepless at night, and frequently cried out as from pain, sweated much, especially about the head. Up to six months she was quite well apparently, weighed 25 pounds, and had rosy cheeks. Shortly after this age, however, she began to show some weakness in sitting, and it was noted that she did not move her legs freely, and that she assumed a queer posture. Then in about a month the gums became swollen, and soon became sore and sometimes bled. About this time the right ankle became slightly swollen, the swelling soon disappearing. This was attributed at the time to rheumatism. Shortly afterwards purpuric-like spots appeared at the position of this swelling and upon the arms, and the child became weaker and weaker, and could not bear to be handled from pain. It was suggested that the weakness and the posture assumed were perhaps due to Pott's disease of the spine; but the sore gums, swollen ankle and purpura led to the belief in the scorbutic nature of the affection, and in consultation this idea was confirmed. The child was placed upon Pasteurized milk (diluted, four ounces of milk to two ounces of water every three hours), and every day the juice of one orange was given in small amounts at a time, as well as one, and afterwards two teaspoonfuls of raw beef-juice. The child was not handled save as necessary. The effect was "magical;" the orange juice was taken from the first with ravenous eagerness, and the meat juice scarcely less greedily. The temperature quickly came to normal, and in a very short time the fretfullness, sleeplessness and other symptoms of irritability disappeared; the gums lost their angry color, and the limbs were moved. Rapid recovery followed. It is not improbable that scurvy is not infrequently overlooked in infants and children, and that more attention should be given the subject.

---

IDIOPATHIC PERITONITIS.—(*Medical Age*, February 25, 1894), Dr. James Sampson, of Windsor, Ontario, after commenting on the address delivered by Dr. Roswell Park, upon peritonitis before the Wayne County Medical Society, records briefly the occurrence of two epidemics of peritonitis. The first occurred near the village of Monpath in 1886. About thirty cases were attacked, most of them suddenly; and the mortality reached about 40 per cent. The suddenness of the attack in the midst of health, the pain and tenderness of the entire abdominal area, the tympanites, nose-bleed, and the fairly definite course (lasting about two weeks), were notable features in these cases. The second epidemic took place in the Essex Centre district. Its extent is not stated, but the impression is given that it was large; in the experience of some physicians, the mortality reached as much as fifty per cent. Marked epistaxis, a sudden and severe onset, a general involvement of the peritoneum, were important features of the disease in the second as in the first epidemic.

---

TREATMENT OF ERYSIPELAS WITH HYPODERMATIC INJECTIONS OF CARBOLIC ACID.—Dr. J. McFadden Gaston, of Atlanta, Ga., (*Medical and Surgical Reporter*, March 24, 1894), recommends, after an extensive experience covering twenty years of use of the method, hypodermatical administration of carbolic acid in erysipelas. He suggests the following formula:

| | | |
|---|---|---|
| ℞ | Carbolic acid | fl ʒ i. |
| | Glycerine | fl ʒ iii. |
| | Distilled water | fl ʒ iv. |

Mix and inject hypodermatically one syringeful in each portion of the size of a hand, daily.

This formula represents a 12½ per cent. solution, and in only a few cases has any local irritation resulted from the injection. This solution is injected at various points over the erysipelatous area, each puncture being sufficient for an area the size of the palm of the hand. Where the surface involved is large, or much fever is present, internal medication is recommended, the best effects being secured by giving calomel at the onset, calomel followed by Epson salts in senna tea, to procure free evacuations from the bowels. After purgation, tincure of iron chloride (gtt. xxv.) is given every three hours until an ounce is taken. Where a phlegmonous form exists chlorate of potash may be combined with the iron in ten grain doses. Where the case is seen early and the inflammation is circumscribed, the writer relies however, upon the hypodermatic use of carbolic acid and rarely has occasion to employ other means of cure.

